# Cardiac Toxicity From Adjuvant Targeting Treatment for Breast Cancer Post-Surgery

**DOI:** 10.3389/fonc.2022.706861

**Published:** 2022-03-24

**Authors:** Zhenkun Fu, Zhoujun Lin, Mao Yang, Chenggang Li

**Affiliations:** ^1^Department of Immunology & Wu Lien-Teh Institute & Heilongjiang Provincial Key Laboratory for Infection and Immunity, Harbin Medical University & Heilongjiang Academy of Medical Science, Harbin, China; ^2^State Key Laboratory of Medicinal Chemical Biology and College of Pharmacy, Nankai University, Tianjin, China; ^3^Basic Medical College, Harbin Medical University, Harbin, China; ^4^Department of Cardiology, The Fourth Affiliated Hospital of Harbin Medical University, Harbin, China

**Keywords:** cardiotoxicity, targeting agents, breast cancer, radiotherapy, chemotherapy

## Abstract

Breast cancer is one of the most prevalent types of cancers worldwide, especially for females. Surgery is the preferred treatment for breast cancer, and various postoperative adjuvant therapies can be reasonably used according to different pathological characteristics, especially traditional radiotherapy, chemotherapy, and endocrine therapy. In recent years, targeting agent therapy has also become one of the selective breast cancer treatment strategies, including anti-HER-2 drugs, CDK4/6 inhibitor, poly ADP-ribose polymerase inhibitor, PI3K/AKT/mTOR pathway inhibitor, ER targeting drugs, and aromatase inhibitor. Because of the different pathologic mechanisms of these adjuvant therapies, each of the strategies may cause cardiotoxicity in clinic. The cardiac adverse events of traditional endocrine therapy, radiotherapy, and chemotherapy for breast cancer have been widely detected in clinic; however, the targeting therapy agents have been paid more attention with the extension of application. This review will summarize the cardiac toxicity of various adjuvant therapies for breast cancer, especially for targeting drug therapy.

## Introduction

Breast cancer is one of the most common malignant tumors in females worldwide, with more than one million new cases per year (1). The main advanced treatment of breast cancer, which is combination of surgery, radiotherapy, chemotherapy, endocrine therapy, and other adjuvant therapies, basically can get to the better outcome and long-term breast cancer survival rate (2). Surgery is considered to be the core basis of breast cancer treatment; other adjuvant therapies were designed to reduce the risk of recurrence and eradicate metastasis. According to the tumor staging, the individual patients’ comorbid condition, the clinicopathological and molecular characteristics of breast cancer, and the appropriate adjuvant therapies were chosen for different breast cancer patients clinically. However, almost each adjuvant therapy may potentially cause acute or chronic clinical adverse effects, such as cardiac complications based on the published articles over the years. Especially, the application of molecular targeting drugs for breast cancer was promoted in recent years; the new therapeutic cardiac toxicity has gradually attracted the rising concern in prognosis of breast cancer.

The aim of this review is to summarize the reported incidences of cardiac toxicity after mainstream breast cancer adjuvant therapies, including myocardial fibrosis, angina, infarction, atrial fibrillation, and ventricular arrhythmia. The focus of this review is to elaborate the association between the clinical adjuvant therapy of breast cancer, such as endocrine therapy, chemotherapy, radiotherapy, targeting therapy, and other treatment rather than radical surgery, and cardiotoxicity as described above. So far, the mechanisms of cardiotoxicity induced by adjuvant therapies for breast cancer have not been clearly described; this review will also provide a brief summary of the probable factors caused by each adjuvant therapy.

## Traditional Adjuvant Therapies and Cardiac Toxicity

Some chemotherapy drugs for breast cancer treatment can induce the cardiac disorders in clinical application according to the reference in recent years. Most chemotherapy drugs include anthracycline, taxanes, cyclophosphamide, cis-platinum, and 5-fluorouracil. According to the side effect of each one, these chemotherapy drugs may lead to the cardiotoxicity for breast cancer patients. In general, there was a clinical threshold for the cumulative dosage of chemotherapy drugs to breast cancer patients, but there is no formal standard to define the threshold of inducing cardiac toxicity in patients until now (**Table 1**). Therefore, we described the cardiotoxicity in breast cancer patients at normal doses of chemotherapeutic agents. Of course, the side effects, including cardiotoxicity, may be more pronounced in patients with dose-dense chemoradiotherapy, such as AC treatment with anthracycline and cyclophosphamide. To avoid serious adverse effects in these patients, rehydration to prevent dehydration and the application of antidotes were commonly used in clinic. However, recent literatures have revealed that the cardiotoxic effects of dose-dense anthracyclines were not as severe as expected (9, 10).

**Table 1:** The threshold/cumulative dose of each chemotherapy drug and radiation for cardiac toxicity.

Drugs for breast cancer treatment (references no.)	Threshold/cumulative dose for cardiac toxicity (incidence of cardiac toxicity%)
Chemotherapy	
Anthracycline-based	
Epirubicin (3)	900 mg/m^2^ (about 1%–11%)
Doxorubicin (3, 4)	500–550 mg/m^2^(about 7%–25%)
Idarubicin (5)	290 mg/m^2^ (about 5%–18%)
Taxanes (4)	135–250 mg/m^2^ and intravenous <3 h (about 25%–55%, almost transient)
Cyclophosphamide (4)	1,500 mg/m^2^ /day (about 20%–40%)
5-Fluorouracil (6)	2,800 mg/m^2^ (about 25%)
Capecitabine (7)	2,500 mg/m^2^/day (about 3%–35%)
Radiotherapy *(* 8*)*	Total 5000 cGy or 650 cGy/one time (hypofractionation) (about 3%–26%)

## Chemotherapies and Cardiotoxicity

The most common anthracycline-based drugs included doxorubicin, epirubicin, daunorubicin, and idarubicin, which have well-documented side effects of cardiac toxicity (11, 12), especially arrhythmias, left ventricular dysfunction, and heart failure (13, 14). Although cardiotoxicity of anthracycline is an accepted side effect in clinic, the clear mechanism remains to be a questionable issue. So far, there are two most likely hypotheses for the anthracycline-induced cardiotoxicity, the damage of oxygen-free radicals, and interaction with the topoisomerase-II-beta enzyme (Top2β) in myocytes (11, 15). As for oxidative stress, anthracyclines can bind to the endothelial cell-specific nitric oxide synthase reductase region, resulting in oxygen free radicals and superoxide compounds increasing and NO synthesis decreasing. When anthracyclines reach a certain concentration in the mitochondria, cationic anthracyclines can also attract negatively charged cardiolipin in the mitochondrial lining, forming irreversible complexes that are also vulnerable to ROS attacks, thus resulting in level changes of Bcl-2 and the pro-apoptotic protein Bax. Cardiolipin peroxidation induced caspase-dependent myocardial cell apoptosis and necrosis. On the other hand, anthracyclines can form a covalent complex with topoisomerase-II, causing double-stranded DNA breakage. Anthracycline can insert into the DNA of cardiomyocytes through the isozyme TOP2β that promotes mitosis of cardiomyocytes, increases the mitochondrial permeability of cardiomyocytes, and leads to acute damage and necrosis of cardiomyocytes (15–17). Anthracycline-related cardiac toxicities represented a common form of chemotherapy adverse effect, and the risk of clinical cardiotoxicity revealed an increasing tendency among breast cancer patients treated with anthracycline-related drugs compared to those treated with non-anthracycline-based regimens by the meta-analysis (18, 19). In order to maximize both quality of life and survival, the aim of balancing the risks of cardiotoxicity and the benefits of antitumor therapy should be paid more attention, especially the understanding of the mechanisms of cardiotoxicity by antitumor therapy (20, 21). Meanwhile, in breast cancer patients with anthracycline-based chemotherapy, it is also essential to take into account other classical risk factors for cardiac toxicity as well, such as age, cumulative dose, administration of other cardiotoxic chemotherapeutic agents, and preexisting cardiovascular disease (3, 5, 22–24). The iron-chelating agent dexrazoxane, applied in the management and treatment of anthracycline-induced cardiotoxicity and extravasation injuries in clinic, is thought to decrease the cardiac adverse effect of anthracycline-based drugs by blocking the generation of free radicals, and in most studies, dexrazoxane did not affect the clinical outcome of anthracycline therapy (25, 26).

Application of taxanes, including paclitaxel and docetaxel, was thought to reduce the risk of recurrence of breast cancer with anthracycline-based chemotherapy as an adjuvant therapy. Taxanes seem to interfere with the metabolism and excretion of anthracycline metabolites leading to potential cardiac toxicity, such as left ventricular diastolic dysfunction and arrhythmias (4). Epothilones, a taxane-like drug, has been applied to the clinical treatment of breast cancers for the past years, but there is not enough data on its cardiotoxicity because of insufficient clinical feedback (27, 28).

Cyclophosphamide, which can inhibit DNA replication and apoptosis, is a kind of DNA-alkylating agent for breast cancer, cyclophosphamide-induced cardiac damage is dose dependent, and mechanisms of cyclophosphamide-induced cardiotoxicity encompass oxidative and nitrative stress and protein adduct formation. The common clinical manifestations of cardiotoxicity include cardiomyocyte inflammation, altered calcium homeostasis, swelling of the cardiomyocytes, and heart failure (4, 29, 30). Because of toxicity of cyclophosphamide, it is limited in clinical application, so further studies on cyclophosphamide cardioprotective antioxidants should be carried out in preclinical studies.

5-Fluorouracil (5-FU) and capecitabine are two common but different ways of administration chemotherapy fluoropyrimidine drugs used to treat breast cancer with similar efficacy. Capecitabine, one kind of oral fluoropyrimidine chemotherapy drug, can be catalyzed to 5-FU by thymidine phosphorylase in tumor tissue. One of the severe side effects to 5-FU and capecitabine-based treatment is various kinds of cardiotoxicity, such as myocardial ischemia, dysarteriotony, left ventricular dysfunction, cardiac arrest, and sudden death (6, 7, 31–36). 5-FU was administered by two ways of oral and intravenous injection in clinic. Oral 5-FU derivatives mainly applied were ftorafur (FT), doxifluridine (5′-DFUR), UFT, carmofur (HCFU), and S-1 (FT and two enzyme inhibitors, 5-chloro-2,4-dihydroxypyridine and potassium oxonate with ratio of 1:0.4:1). Due to irregular absorption of oral 5-FU, the main way of administration in clinic was intravenous injection, so the cardiotoxicity of 5-FU was mostly caused by intravenous injection in breast cancer treatment; only few patients showed myocardial ischemia adverse effects with oral fluoropyrimidine drugs. Moreover, capecitabine has a cardiac toxicity similar to intravenous 5-FU, such as myocardial infarction and coronary vasospasm (37, 38). The two most likely mechanisms of 5-FU-related cardiotoxicity are ischemia and drug-related myocardial toxicity. Coronary vasospasm is one of likely leading theories for 5-FU-related myocardial ischemia by endothelial dysfunction or smooth muscle dysfunction, and according to these hypotheses 5-FU may lead to thrombotic occlusive disease (39–41). The pathological mechanisms that lead to 5-Fu-induced cardiotoxicity also include oxidative stress and direct cell damage, but these mechanisms are limited to experimental models (42–44). In human autopsy subjects, ventricular dilatation and scatter necrosis with an inflammatory infiltrate and proliferation of the sarcoplasmic reticulum were also demonstrated in 5-FU treatment patients (45). According to these hypotheses, 5-FU-induced cardiotoxicity might be multifactorial; further research is needed to clarify the pathogenesis of these adverse effects.

## Radiotherapy and Cardiac Toxicity

Radiotherapy is an indispensable unit of multidisciplinary treatment of breast cancer; it can reduce mortality and the risk of local recurrences by about two-thirds (46). However, as the adjacent organ, cardiac irradiation damage was unintentional in breast cancer patients with radiotherapy. Radiotherapy was recognized as the common factor of cardiac mortality among breast cancer survivors, with a maximum increase of nearly 1.7 times in cardiovascular mortality from long-term radiotherapy compared with surgery, and the risk increased over time (8, 47). The pathophysiological mechanisms of radiation-induced cardiac disease are related to vascular damage, which may lead to pericarditis, coronary artery disease, acute myocardial infarction, cardiomyopathy, or valvular heart disease (48, 49). However, valvular heart disease rarely occurred on radiation for conserved breast or post-mastectomy radiation therapy, and only few patients developed symptoms 5 to 10 years after radiotherapy (50). Similar to chemotherapy, radiation therapy also has the relatively safe threshold of the cumulative amount of radioactive substances, exceeding the cumulative dose that may increase the risk of cardiotoxicity, but there is no unified standard for this threshold now (**Table 1**). Also, except for radiation dose, the risk of cardiac toxicity for breast cancer radiotherapy was associated with radiation time and higher abnormalities after left-breast irradiation compared with the right (51–54).

Considering the benefit of radiation therapy in breast cancer treatment and improving survival, clinicians should pay more attention to the development of cardiovascular disease in patients. New radiation administration techniques, including cardiac field shielding and radiation dose reduction, may decrease the risk of developing cardiac disease (55). Recently, in order to reduce the cardiac damage of irradiation, intensity-modulated radiotherapy (IMRT) has been applied in clinical breast cancer treatment, including rotational and proton IMRT (56, 57). In addition, so as to improve the effect of target coverage and decrease the risk of cardiac dose in breast cancer radiation patients, new radiation therapies such as deep-inspiration breath hold (DIBH) and multi-leaf collimators (MLC) have also been applied in clinic (54, 58, 59).

Based on the advantages of radiotherapy in prolongation of survival in breast cancer patients, the abandoning of radiation therapy was not recommended due to cardiotoxicity. So the risk of breast cancer-specific mortality and the cardiac risk factors must be weighed up against the risk of radiation-induced cardiotoxicity, respectively. Taking these factors into consideration, the application of radiotherapy should consider the age and pathological grade of patients; the individual decision between heart protection and optimal target coverage was still up to the physicians (48, 60, 61).

## Targeting Drugs and Cardiac Toxicity

In recent years, targeting drugs for tumor treatment became the clinical hotspot including breast cancer therapy (62). There were several hot targeting drugs in breast cancer treatment, comprising the HER-2-targeting monoclonal antibody (mAb), CDK4/6 inhibitor, poly ADP-ribose polymerase (PARP) inhibitor, PI3K/AKT/mTOR pathway inhibitor, ER targeting drugs, and aromatase inhibitor (AI). For these targeting pharmaceuticals, the most common category of targeting drugs is the class of humanized anti-HER-2 mAb, especially for HER-2-positive breast cancer treatment.

## Anti-Her-2 Targeting Drugs Induced Cardiotoxicity

Human epidermal growth factor receptor-2 (HER-2) is a membrane tyrosine kinase receptor, together with HER-1 (also called EGFR), HER-3, and HER-4, to form the EGFR family. No ligand is known for HER-2 now, but ligand-stimulated HER-1, HER-3, and HER-4 forming homodimers or combined with HER-2 in heterodimers can elicit a series of physiological cellular responses. For HER-2-positive breast cancer, the formation of heterodimers with HER-1 and HER-3 leads to the activation of signaling pathways promoting proliferation and survival of cancer cells (63).

With the extensive application of HER-2-targeting drugs in clinical HER-2-positive breast cancer patients, trastuzumab can improve the poor prognosis and prevent metastasis, and it even has a significant effect on improving survival among the adjuvant therapies (64–66). Since late 1990s, trastuzumab was first applied for HER-2-positive and metastatic breast cancer. Trastuzumab can bind to subdomain IV of the extracellular domain of the receptor. Following the binding with the receptor, trastuzumab can disrupt the ligand-independent interaction of HER-2/HER3/PI3K complex downstream signaling, strengthen the activity of cell-cycle inhibitor p27 potentially, inhibit the growth of breast cancer, and cause the death of cancer cells by antibody-dependent immune cell-mediated cytotoxicity (67–69). However, the cardiotoxicity of trastuzumab was reported within a few years, and even the incidences of cardiac dysfunction were up to unbelievably close 30% by combining with chemotherapy (70). According to the clinical indication, the targeting adjuvant trials for breast cancer must choose strictly in accordance with cardiac exclusion criteria, monitoring of cardiac function, cardiac safety analysis, and appropriate administration of trastuzumab combined with anthracycline-based chemotherapy drugs (71, 72).

Although trastuzumab has the notable effect on HER-2-positive breast cancer, it also causes various significant adverse reactions including cardiac dysfunction. The left ventricular (LV) dysfunction and congestive heart failure were the most common cardiotoxicity disorders with the duration of drugs, especially combined with anthracycline-based chemotherapy treatment at the same time (73–76). However, these disorders could be medically controlled by interval administration or cardiac protective drugs (77, 78), so trastuzumab could be used clinically.

Following the development of targeting drugs, another FDA-approved anti-HER-2 humanized monoclonal antibody pertuzumab came out. Compared with trastuzumab, pertuzumab also targets the extracellular portion of HER-2, but at a different epitope of subdomain II (79). Pertuzumab also triggers antibody-dependent cell-mediated cytotoxicity, but it prevents ligand-initiated heterodimerization of HER-2 and HER-3 (80). Although trastuzumab and pertuzumab recognize different sites of HER-2, their clinical combination results in stronger antitumor efficacy (81). Surprisingly, the combination of pertuzumab and trastuzumab does not seem to raise the risk of LV dysfunction and congestive heart failure and even alleviate the left ventricular dysfunction possibly (82–84). The latest recombinant HER-2 humanized monoclonal antibody inetetamab has entered into the clinical treatment of breast cancer in the past 2 years, and so far it only seems to cause the decrease of LVEF.

Due to the late application of HER-2-targeting therapy compared with traditional chemotherapy drugs, breast cancer patients were treated with HER-2-targeting drugs later than conventional chemotherapy drugs, such as anthracyclines. Lapatinib, a dual EGFR/HER-2 tyrosine kinase inhibitor, has been found to exert significant biologic effects on the inhibition of signaling pathways to promote breast cancer cell proliferation and survival, especially in terminal, recurrent, or metastatic breast cancer patients who had experienced anthracyclines treatment (85). According to the collection of the clinical trial data in recent years, lapatinib may have the lower cardiotoxicity than pertuzumab and trastuzumab, especially for congestive heart failure (86–88). However, there is still uncertainty on the cardiac toxicity of lapatinib (89). Using the neonatal rat cardiac myocyte model, chemotherapy-induced myocyte damage was greatly enhanced by the addition of nanomolar lapatinib concentrations, although lapatinib treatment alone only slightly induced myocyte damage. Treatment by lapatinib alone decreased phosphorylated ERK (MAPK), which may have increased myocyte damage. Moreover, lapatinib is a strong inhibitor of several ATP-dependent ABC-type efflux transporters, so lapatinib may block doxorubicin efflux to increase intracellular doxorubicin concentrations leading to myocyte damage (90). Another tyrosine kinase inhibitor neratinib, which is an intensive adjuvant drug for trastuzumab for early-stage HER-2-positive breast cancer patients, has insufficient evidence of cardiac adverse effect, but only causing diarrhea (91).

There is no clear definition on the cardiotoxicity mechanisms of HER-2-targeting drugs now. According to the analysis of myocardial deformation indexes by speckle tracking echocardiography, impairment in apical rotation was observed. It seems to be the first sign of global left ventricular (LV) dysfunction predicting global longitudinal strain reduction during trastuzumab treatment (92). Because of the few histological correlates and the cardiac symptoms disappearing after withdrawal, HER-2-targeting mAb is commonly viewed as transient. So there were few breast cancer patients that showed LV function damage (73, 93). Cardiac progenitor cells ensure the limited capability of heart regeneration following injury, and the HER-2-targeting drugs have the ability to hinder the cardiomyogenic and angiogenic capacities of cardiac graft enriched for these cells (94). Some studies have pointed out that neuregulin-1 (NRG-1) may play a major role in the formation of cardiac toxicity in HER-2-targeting treatment (95). NRG-1 is the ligand of HER-4; NRG-1 can induce the protective response of cardiomyocyte stress by binding of HER-4/HER-4 homodimers and HER-4/HER-2 heterodimers in animal models and cell culture studies. Moreover, cardiomyocytes from *Erbb-2* knockout mice are more susceptible to cause chemotherapy toxicity of myofibrillar disarray. According to the above mechanism, HER-2-targeting mAb antagonists may impede the formation of NRG-1-triggered HER-4/HER-2 heterodimers to produce cardiac damage (96). The emergence of some doubts, such as HER-2-targeting therapy for downregulation of HER-2 in the mouse cardiac tissue instead of cancer cells (97, 98) and the validity of the NRG-1/HER-2 paradigm (99), suggests that there are still unknown specific mechanisms to explore in the formation of cardiotoxicity by HER-2-targeting therapy. Due to that traditional chemotherapy drugs and targeted drugs have certain cardiac toxicity in breast cancer treatment, the combination application of anthracyclines and HER-2-targeted therapy seems to significantly increase vascular endothelial dysfunction compared with targeted therapy alone. However, the predictive effect of the combination of anthracyclines and HER-2-targeted therapy on cardiotoxicity in breast cancer patients remains to be confirmed (100). So breast cancer patients should try to avoid the combined application of HER-2-targeting and anthracycline therapy, or apply beta-blockers for protection.

## CDK4/6 Inhibitors Induced Cardiotoxicity

Cyclin-dependent kinase (CDK) family members interact with cyclin D proteins to play an important role in cell-cycle progression, so the CDK family represents a potential target for tumors including breast cancer. Cyclin-dependent kinases 4 and 6 (CDK4/6) in complex with cyclin D subunits phosphorylated the antiproliferative retinoblastoma (Rb), which can regulate the progression of the cell cycle by binding with the E2F family of transcription factors, allowing increased synthesis of genes importantly for DNA replication and thus progression from Phases G1 to S of the cell cycle (101, 102). Because inhibitors of CDK4/6 have the function of blocking the proliferation of tumor cells, they have been persistent in clinical trial development for the treatment of ER-positive and HER-2-negative breast cancer in the last decade. The typical breast cancer-targeting drugs of CDK4/6 inhibitors include palbociclib, ribociclib, and abemaciclib.

Palbociclib and ribociclib, in combination with other adjuvant treatments for advanced or metastatic breast cancer, are CDK inhibitors with a similar mechanism. Palbociclib tends to apply in hormone receptor-positive and HER-2-negative breast cancer patients. These two agents have shown different cardiotoxic effects with unclear mechanisms. In particular, ribociclib has been associated with QT interval prolongation, but not palbociclib (103). However, in a recent mouse model study, palbociclib was found to protect cardiac tissue from necrosis, localized fibrosis, and hypertrophy of cardiomyocytes in diabetic cardiomyopathy interestingly (104). As a new CDK4/6 inhibitor to metastatic breast cancer treatment for about 3 years, abemaciclib does not have sufficient clinical feedback on cardiotoxicity.

## PARP and PI3K/AKT/mTOR Pathway Inhibitors Induced Cardiotoxicity

Poly ADP-ribose polymerases (PARP) are a group of DNA damage-repairing enzymes. PARP1/2 assists in the repair of single-strand breaks through base excision repair. Inhibition of PARP results in the trapping of the PARP–DNA complex at replication forks, causing single-strand breaks to become double-strand breaks (105). Tumor-suppressor gene *brca1/2* accounts for about 10% of breast cancer cases, and the lifetime risk of developing breast cancer in *brca1/2* mutation carriers is about 70% (106). BRCA1/2 are responsible for the repair of double-strand DNA breaks; deficiency of BRCA1/2 is particularly sensitive to the effects of PARP inhibition owing to repair double-strand break dysfunction. The PARP inhibitor olaparib has been applied in HER-2-negative, metastatic breast cancer with a *brca1/2* mutation for only about 10 years (107). The adverse effect data were limited due to the short duration of olaparib clinical application, including cardiotoxicity. According to the recent studies, no cardiotoxicity of olaparib was found, such as heart failure or QT/QTc interval (108). However, in a rat model, olaparib protected cardiomyocytes against oxidative stress and improved graft contractility in heart transplantation, and this may show the possibility of tumor-targeting drug olaparib for heart transplantation (109).

The PI3K/AKT/mTOR pathway can regulate cell growth, survival, and proliferation. The mammalian target of rapamycin (mTOR) is a key modulator of signals governing protein and lipid biosynthesis and cell-cycle progression, so mTOR can drive cancer growth by activating the lipid and protein biosynthesis (110). The mTOR inhibitor everolimus (Afinitor), a derivative of rapamycin, binds with high affinity to its intracellular receptor FKBP12. The everolimus–FKBP12 complex inhibits mTOR to prevent the downstream signaling required for cell-cycle progression, cell growth, and proliferation (111). Everolimus is therefore used for mTOR-positive, hormone receptor-positive, and HER-2-negative advanced breast cancer in clinic to restore hormone sensitivity. In limited studies of everolimus to breast cancer, there were no serious adverse effects treated with everolimus so far, such as acute coronary events, arrhythmias, acute heart failure, and left ventricle ejection fraction. However, other metabolic syndromes, hyperglycemia and hyperlipidemia, associated with cardiotoxicity were reported, so it may lead to a risk of aggravating atherosclerosis (112).

## ER-Targeting Drugs Induced Cardiotoxicity

Tamoxifen (Nolvadex), an estradiol competitive modulator, can bind with ER to deactivate the transcription for early or advanced breast cancer (113). The most common cardiac adverse effect of tamoxifen was ischemic heart disease in clinic (114). However, according to the modulating lipid metabolism function of tamoxifen, tamoxifen may decrease the risk of cardiovascular disease, but it was not shown to have any benefit on cardiovascular risk in breast cancer treatment (115–117). Toremifene citrate, a selective estrogen receptor modulator, was developed in the 1990s with the efficacy similar to tamoxifen and with an improved safety profile. During these years, a number of studies have investigated the association between toremifene citrate and cardiovascular mortality, but it is still unclear whether the relationship exists (118). Fulvestrant, a highly selective estrogen receptor downregulator for postmenopausal HR^+^/HER-2^-^ advanced breast cancer patients by injection, can bind with ER and accelerate its degradation (119). Fulvestrant is extremely well tolerated with rare occurrence of ischemic cardiovascular disorders, atrial tachycardia, and cardiac failure adverse effects (120–122).

## Aromatase Inhibitors Induced Cardiotoxicity

Estrogen in postmenopausal women relies on aromatase enzymes to convert androgen from the adrenal cortex into estrogen. Aromatase inhibitors (AI), including anastrozole, letrozole, and exemestane, acted as first-line endocrine therapy for advanced breast cancer in postmenopausal women. Anastrozole and letrozole are two reversible aromatase inhibitors; the irreversible aromatase inactivator was exemestane.

Letrozole and anastrozole can reduce plasma estrogen levels for advanced breast cancer in postmenopausal women who cannot be controlled by anti-estrogen therapies. According to clinical data, blood lipids seem to be increased during therapy with AI (anastrozole, letrozole, and exemestane), leading to a series of cardiac adverse events probably by hypercholesterolemia (123–126). Moreover, in real-time PCR analysis for rats models, letrozole can induce significant changes in renin–angiotensin system-related genes to result in cardiac events (127).

However, the AI application rarely led to significant cardiac adverse events in clinical treatment over the years. In the treatment with trastuzumab plus anastrozole, only one patient experienced congestive heart failure (128). In rare cases, anastrozole could cause myocardial infarction independent of treatment time (129).

## Conclusion

Chemotherapy and radiotherapy are important adjuvant treatments for breast cancer patients, and new treatment strategies targeting HER-2, CDK4/6, ER, or aromatase have appeared in recent years. With the increase in clinical application, these treatment strategies were found to be associated with cardiac adverse effects through different pathophysiological mechanisms (**Table 2**). Compared with other adjuvant treatments of breast cancer, more and more newly found targeting agents for breast cancer were applied in clinics with less cardiotoxicity. However, it is also necessary to provide appropriate cardiac monitoring and initiation of cardiovascular medication to reveal cardiac dysfunction.

**Table 2:** The published potential molecular biological mechanisms of cardiotoxicity for breast cancer adjuvant treatment.

Drugs for breast cancer treatment	Potential molecular mechanisms of cardiac toxicity	References
**Chemotherapy drugs**		
*Anthracycline (epirubicin, daunorubicin, idarubicin)*	a. The damage of oxygen free radicals. b. interaction with the topoisomerase-II-beta enzyme (Top2β) in myocytes	(11, 15–17, 25)
*Taxanes*	Interfere with the metabolism and excretion of anthracycline	(28)
*Cyclophosphamide*	a. Oxidative and nitrative stress. b. Protein adduct formation.	(29, 30)
*5-Fluorouracil and capecitabine*	Vasospasm and thrombosis. Oxidative stress	(36, 39–43)
**Radiotherapy**	Vascular damage and higher abnormalities after left-breast irradiation	(46, 48, 49)
**Targeting drugs**		
*Anti-Her-2 targeting drugs (trastuzumab, pertuzumab, lapatinib)*	a. May decrease phosphorylated ERK (MAPK) and increase intracellular doxorubicin concentrations. b. Hinder the cardiomyogenic and angiogenic capacities. c. Neuregulin-1 (NRG-1) bind with HER-4	(89, 90, 94–96)
*CDK4/6 inhibitors (palbociclib, ribociclib, abemaciclib)*	Unclear	
*PARP and PI3K/AKT/mTOR pathway inhibitors (olaparib, rapamycin, everolimus)*	Aggravating atherosclerosis, hyperglycemia, and hyperlipidemia.	(112)
		
*ER targeting drugs (tamoxifen, toremifene citrate, fulvestrant)*	Ischemic disorder.	(115, 121)
*AI (anastrozole, letrozole, exemestane)*	a. Causing hypercholesterolemia. b. Renin–angiotensin system related genes changing	(123–127)

Guidelines for the monitoring and management of cancer treatment-induced cardiotoxicity are available from the American Society of Clinical Oncology (ASCO) and the European Society of Cardiology (ESC) (130, 131). We have seen a number of patients with heart disorders after breast cancer treatment in clinic. Thus, the treatment plan for each individual breast cancer patient must be carefully considered to choose both the appropriate therapy and the necessary cardiac monitoring plan. As cardiotoxicity of breast cancer therapy is well recognized, there was little evidence for the treatment of patients with baseline cardiac diseases in clinic. Clinically, breast cancer patients with cardiac dysfunction and arrhythmias are often monitored and intervened during the treatment, such as application of angiotensin-converting-enzyme (ACE) inhibitors or beta-blockers. Therefore, collaboration among pharmacologists, cardiologists, and oncologists in both scientific research and clinical trial is important to develop cardioprotective strategies for breast cancer patients.

## Author Contributions

All the authors equally contributed to the preparation of this manuscript. All authors contributed to the article and approved the submitted version.

## Funding

This work was supported by the Postdoctoral Science Foundation initially funded project of Heilongjiang Province (LBH-Q19153) and the Funds on Health Commission of Heilongjiang Province (2016-120), China.

## Conflict of Interest

The authors declare that the research was conducted in the absence of any commercial or financial relationships that could be construed as a potential conflict of interest.

## Publisher’s Note

All claims expressed in this article are solely those of the authors and do not necessarily represent those of their affiliated organizations, or those of the publisher, the editors and the reviewers. Any product that may be evaluated in this article, or claim that may be made by its manufacturer, is not guaranteed or endorsed by the publisher.

## References

[1] DeSantis CE Ma J Gaudet MM Newman LA Miller KD Sauer AG. Breast Cancer Statistics 2019. CA Cancer J Clin (2019) 69:438–51. doi: 10.3322/caac.21583 31577379

[2] Siegel R Naishadham D Jemal A. Cancer Statistics, 2012. CA Cancer J Clin (2012) 62:10–29. doi: 10.3322/caac.20138 22237781

[3] Mantovani G Madeddu C Cadeddu C Dessi M Piras A Massa E. Persistence, Up to 18 Months of Follow-Up, of Epirubicin-Induced Myocardial Dysfunction Detected Early by Serial Tissue Doppler Echocardiography: Correlation With Inflammatory and Oxidative Stress Markers. Oncologist (2008) 13:1296–305. doi: 10.1634/theoncologist.2008-0151 19060235

[4] Morandi P Ruffini PA Benvenuto GM Raimondi R Fosser V. Cardiac Toxicity of High-Dose Chemotherapy. Bone Marrow Transplant (2005) 35:323–34. doi: 10.1038/sj.bmt.1704763 15543194

[5] Anderlini P Benjamin RS Wong FC Kantarjian HM Andreeff M Kornblau SM. Idarubicin Cardiotoxicity: A Retrospective Study in Acute Myeloid Leukemia and Myelodysplasia. J Clin Oncol (1995) 13:2827–34. doi: 10.1200/JCO.1995.13.11.2827 7595745

[6] Peng J Dong C Wang C Li W Yu H Zhang M. Cardiotoxicity of 5-Fluorouracil and Capecitabine in Chinese Patients: A Prospective Study. Cancer Commun (2018) 38:22. doi: 10.1186/s40880-018-0292-1 PMC5953402 29764506

[7] Chua YJ Barbachano Y Cunningham D Oates JR Brown G Wotherspoon A. Neoadjuvant Capecitabine and Oxaliplatin Before Chemoradiotherapy and Total Mesorectal Excision in MRI-Defined Poor-Risk Rectal Cancer: A Phase 2 Trial. Lancet Oncol (2010) 11:241–8. doi: 10.1016/S1470-2045(09)70381-X 20106720

[8] Darby SC McGale P Taylor CW Peto R. Long-Term Mortality From Heart Disease and Lung Cancer After Radiotherapy for Early Breast Cancer: Prospective Cohort Study of About 300,000 Women in US SEER Cancer Registries. Lancet Oncol (2005) 6:557–65. doi: 10.1016/S1470-2045(05)70251-5 16054566

[9] Swain SM Ewer MS Viale G Delaloge S Ferrero JM Verrill M. Pertuzumab, Trastuzumab, and Standard Anthracycline- and Taxane-Based Chemotherapy for the Neoadjuvant Treatment of Patients With HER-2-Positive Localized Breast Cancer (BERENICE): A Phase II, Open-Label, Multicenter, Multinational Cardiac Safety Study. Ann Oncol (2018) 29:646–53. doi: 10.1093/annonc/mdx773 PMC5888999 29253081

[10] Dent SF Botros J Rushton M Aseyev O Levine MN Parulekar WR. Anthracycline-Induced Cardiotoxicity in Patients With Early-Stage Breast Cancer: The Canadian Cancer Trials Group (CCTG) Ma.21 Experience. Breast Cancer Res Treat (2020) 184:733–41. doi: 10.1007/s10549-020-05887-w 32940847

[11] Singal PK Iliskovic N. Doxorubicin-Induced Cardiomyopathy. N Engl J Med (1998) 339:900–5. doi: 10.1056/NEJM199809243391307 9744975

[12] Zhang S Liu X Bawa-Khalfe T Lu LS Lyu YL Liu LF. Identification of the Molecular Basis of Doxorubicin-Induced Cardiotoxicity. Nat Med (2012) 18:1639–42. doi: 10.1038/nm.2919 23104132

[13] Nagy AC GulAcsi-BArdos P CserEp Z Hangody L Forster T. Late Cardiac Effect of Anthracycline Therapy in Physically Active Breast Cancer Survivors - A Prospective Study. Neoplasma (2017) 64:92–100. doi: 10.4149/neo_2017_111 27881009

[14] Schneider BP Shen F Gardner L Radovich M Li L Miller KD. Genome-Wide Association Study for Anthracycline-Induced Congestive Heart Failure. Clin Cancer Res (2017) 23:43–51. doi: 10.1158/1078-0432.CCR-16-0908 27993963 PMC5215621

[15] Deng S Yan T Jendrny C Nemecek A Vincetic M Gödtel-Armbrust U. Dexrazoxane may Prevent Doxorubicin-Induced DNA Damage *via* Depleting Both Topoisomerase II Isoforms. BMC Cancer (2014) 14:842. doi: 10.1186/1471-2407-14-842 25406834 PMC4242484

[16] Spallarossa P Garibaldi S Altieri P Fabbi P Manca V Nasti S. Carvedilol Prevents Doxorubicin-Induced Free Radical Release and Apoptosis in Cardiomyocytes *In Vitro* . J Mol Cell Cardiol (2004) 37:837–46. doi: 10.1016/j.yjmcc.2004.05.024 15380674

[17] Cernecka H Doka G Srankova J Pivackova L Malikova E Galkova K. Ramipril Restores Pparβ/δ and Pparγ Expressions and Reduces Cardiac NADPH Oxidase But Fails to Restore Cardiac Function and Accompanied Myosin Heavy Chain Ratio Shift in Severe Anthracycline-Induced Cardiomyopathy in Rat. Eur J Pharmacol (2016) 791:244–53. doi: 10.1016/j.ejphar.2016.08.040 27592051

[18] Ewer MS Lippman SM. Type II Chemotherapy-Related Cardiac Dysfunction: Time to Recognize a New Entity. J Clin Oncol (2005) 23:2900–2. doi: 10.1200/JCO.2005.05.827 15860848

[19] Smith LA Cornelius VR Plummer CJ Levitt G Verrill M Canney P. Cardiotoxicity of Anthracycline Agents for the Treatment of Cancer: Systematic Review and Meta-Analysis of Randomised Controlled Trials. BMC Cancer (2010) 10:337. doi: 10.1186/1471-2407-10-337 20587042 PMC2907344

[20] Ewer MS Ewer SM. Cardiotoxicity of Anticancer Treatments: What the Cardiologist Needs to Know. Nat Rev Cardiol (2010) 7:564–75. doi: 10.1038/nrcardio.2010.121 20842180

[21] Ewer MS Ewer SM. Cardiotoxicity of Anticancer Treatments. Nat Rev Cardiol (2015) 12:547–58. doi: 10.1038/nrcardio.2015.65 25962976

[22] Lotrionte M Biondi-Zoccai G Abbate A Lanzetta G D’Ascenzo F Malavasi V. Review and Meta-Analysis of Incidence and Clinical Predictors of Anthracycline Cardiotoxicity. Am J Cardiol (2013) 112:1980–4. doi: 10.1016/j.amjcard.2013.08.026 24075281

[23] Ryberg M Nielsen D Cortese G Nielsen G Skovsgaard T Andersen PK. New Insight Into Epirubicin Cardiac Toxicity: Competing Risks Analysis of 1097 Breast Cancer Patients. J Natl Cancer Inst (2008) 100:1058–67. doi: 10.1093/jnci/djn206 18664656

[24] Capri G Tarenzi E Fulfaro F Gianni L. The Role of Taxanes in the Treatment of Breast Cancer. Semin Oncol (1996) 23:68–75. doi: 10.1002/9780470695135.ch17 8614849

[25] van Dalen EC van der Pal HJ Caron HN Kremer LC. Different Dosage Schedules for Reducing Cardiotoxicity in Cancer Patients Receiving Anthracycline Chemotherapy. Cochrane Database Syst Rev (2009) 4:CD005008. doi: 10.1002/14651858.CD005008.pub3 17054232

[26] Wiseman LR Spencer CM. Dexrazoxane. A Review of its Use as a Cardioprotective Agent in Patients Receiving Anthracycline-Based Chemotherapy. Drugs (1998) 56:385–403. doi: 10.2165/00003495-199856030-00009 9777314

[27] Shimoyama M Murata Y Sumi KI Hamazoe R Komuro I. Docetaxel Induced Cardiotoxicity. Heart (2001) 86:219. doi: 10.1136/heart.86.2.219 PMC1729847 11454849

[28] Gianni L Salvatorelli E Minotti G. Anthracycline Cardiotoxicity in Breast Cancer Patients: Synergism With Trastuzumab and Taxanes. Cardiovasc Toxicol (2007) 7:67–71. doi: 10.1007/s12012-007-0013-5 17652806

[29] Iqubal A Iqubal MK Sharma S Ansari MA Najmi AK Ali SM. Molecular Mechanism Involved in Cyclophosphamide-Induced Cardiotoxicity: Old Drug With a New Vision. Life Sci (2019) 218:112–31. doi: 10.1016/j.lfs.2018.12.018 30552952

[30] Ayza MA Zewdie KA Tesfaye BA Wondafrash DZ Berhe AH. The Role of Antioxidants in Ameliorating Cyclophosphamide-Induced Cardiotoxicity. Oxid Med Cell Longev (2020) 2020:4965171. doi: 10.1155/2020/4965171 32454939 PMC7238386

[31] Kosmas C Kallistratos MS Kopterides P Syrios J Skopelitis H Mylonakis N. Cardiotoxicity of Fluoropyrimidines in Different Schedules of Administration: A Prospective Study. J Cancer Res Clin Oncol (2008) 134:75–82. doi: 10.1007/s00432-007-0250-9 17636329 PMC12161746

[32] Polk A Vaage-Nilsen M Vistisen K Nielsen DL. Cardiotoxicity in Cancer Patients Treated With 5-Fluorouracil or Capecitabine: A Systematic Review of Incidence, Manifestations and Predisposing Factors. Cancer Treat Rev (2013) 39:974–84. doi: 10.1016/j.ctrv.2013.03.005 23582737

[33] Ng M Cunningham D Norman AR. The Frequency and Pattern of Cardiotoxicity Observed With Capecitabine Used in Conjunction With Oxaliplatin in Patients Treated for Advanced Colorectal Cancer (CRC). Eur J Cancer (2005) 41:1542–6. doi: 10.1016/j.ejca.2005.03.027 15978800

[34] Meyer CC Calis KA Burke LB Walawander CA Grasela TH. Symptomatic Cardiotoxicity Associated With 5-Fluorouracil. Pharmacotherapy (1997) 17:729–36. doi: 10.1002/j.1875-9114.1997.tb03748.x 9250550

[35] Sara JD Kaur J Khodadadi R Rehman M Lobo R Chakrabarti S. 5-Fluorouracil and Cardiotoxicity: A Review. Ther Adv Med Oncol (2018) 10:1758835918780140. doi: 10.1177/1758835918780140 29977352 PMC6024329

[36] Sudhoff T Enderle MD Pahlke M Petz C Teschendorf C Graeven U. 5-Fluorouracil Induces Arterial Vasocontractions. Ann Oncol (2004) 15:661–4. doi: 10.1093/annonc/mdh150 15033676

[37] Molteni LP Rampinelli I Cergnul M Scaglietti U Paino AM Noonan DM. Capecitabine in Breast Cancer: The Issue of Cardiotoxicity During Fluoropyrimidine Treatment. Breast J (2010) 16 Suppl 1:S45–8. doi: 10.1111/j.1524-4741.2010.01004.x 21050310

[38] Polk A Shahmarvand N Vistisen K Vaage-Nilsen M Larsen FO Schou M. Incidence and Risk Factors for Capecitabine-Induced Symptomatic Cardiotoxicity: A Retrospective Study of 452 Consecutive Patients With Metastatic Breast Cancer. BMJ Open (2016) 6:e012798. doi: 10.1136/bmjopen-2016-012798 PMC5073470 27798021

[39] Jensen SA Sorensen JB. 5-Fluorouracil-Based Therapy Induces Endovascular Injury Having Potential Significance to Development of Clinically Overt Cardiotoxicity. Cancer Chemother Pharmacol (2012) 69:57–64. doi: 10.1007/s00280-011-1669-x 21603868

[40] Spasojevic I Jelic S Zakrzewska J Bacic G. Decreased Oxygen Transfer Capacity of Erythrocytes as a Cause of 5-Fluorouracil Related Ischemia. Molecules (2008) 14:53–67. doi: 10.3390/molecules14010053 19127237 PMC6253945

[41] Lamberti M Porto S Marra M Zappavigna S Grimaldi A Feola D. 5-Fluorouracil Induces Apoptosis in Rat Cardiocytes Through Intracellular Oxidative Stress. J Exp Clin Cancer Res (2012) 31:60. doi: 10.1186/1756-9966-31-60 22812382 PMC3461434

[42] Durak I Karaayvaz M Kavutcu M Cimen MY Kaçmaz M Büyükkoçak M. Reduced Antioxidant Defense Capacity in Myocardial Tissue From Guinea Pigs Treated With 5-Fluorouracil. J Toxicol Environ Health A (2000) 59:585–9. doi: 10.1080/009841000156709 10777249

[43] Kinhult S Albertsson M Eskilsson J Cwikiel M. Effects of Probucol on Endothelial Damage by 5-Fluorouracil. Acta Oncol (2003) 42:304–8. doi: 10.1080/02841860310004409 12899501

[44] Kuropkat C Griem K Clark J Rodriguez ER Hutchinson J Taylor SG. Severe Cardiotoxicity During 5-Fluorouracil Chemotherapy: A Case and Literature Report. Am J Clin Oncol (1999) 22:466–70. doi: 10.1097/00000421-199910000-00009 10521060

[45] Early Breast Cancer Trialists’ Collaborative Group (EBCTCG) Darby S McGale P Correa C Taylor C Arriagada R. Effect of Radiotherapy After Breast-Conserving Surgery on 10-Year Recurrence and 15-Year Breast Cancer Death: Meta-Analysis of Individual Patient Data for 10,801 Women in 17 Randomised Trials. Lancet (2011) 378:1707–16. doi: 10.1016/S0140-6736(11)61629-2 PMC3254252 22019144

[46] Lancellotti P Nkomo VT Badano LP Bergler-Klein J Bogaert J Davin L. Expert Consensus for Multi-Modality Imaging Evaluation of Cardiovascular Complications of Radiotherapy in Adults: A Report From the European Association of Cardiovascular Imaging and the American Society of Echocardiography. J Am Soc Echocardiogr (2013) 26:1013–32. doi: 10.1016/j.echo.2013.07.005 23998694

[47] Hooning MJ Aleman BM van Rosmalen AJ Kuenen MA Klijn JG van Leeuwen FE. Cause-Specific Mortality in Long-Term Survivors of Breast Cancer: A 25-Year Follow-Up Study. Int J Radiat Oncol Biol Phys (2006) 64:1081–91. doi: 10.1016/j.ijrobp.2005.10.022 16446057

[48] Darby SC Ewertz M McGale P Bennet AM Blom-Goldman U Brønnum D. Risk of Ischemic Heart Disease in Women After Radiotherapy for Breast Cancer. N Engl J Med (2013) 368:987–98. doi: 10.1056/NEJMoa1209825 23484825

[49] Baillet F Housset M Maylin C Boisserie G Bettahar R Delanian S. The Use of a Specific Hypofractionated Radiation Therapy Regimen Versus Classical Fractionation in the Treatment of Breast Cancer: A Randomized Study of 230 Patients. Int J Radiat Oncol Biol Phys (1990) 19:1131–3. doi: 10.1016/0360-3016(90)90216-7 2254102

[50] Carlson RG Mayfield WR Normann S Alexander JA. Radiation-Associated Valvular Disease. Chest (1991) 99:538–45. doi: 10.1378/chest.99.3.538 1995208

[51] Nilsson G Holmberg L Garmo H Duvernoy O Sjögren I Lagerqvist B. Distribution of Coronary Artery Stenosis After Radiation for Breast Cancer. J Clin Oncol (2012) 30:380–6. doi: 10.1200/JCO.2011.34.5900 22203772

[52] Correa CR Litt HI Hwang WT Ferrari VA Solin LJ Harris EE. Coronary Artery Findings After Left-Sided Compared With Right-Sided Radiation Treatment for Early-Stage Breast Cancer. J Clin Oncol (2007) 25:3031–7. doi: 10.1200/JCO.2006.08.6595 17634481

[53] Taylor CW Nisbet A McGale P Darby SC. Cardiac Exposures in Breast Cancer Radiotherapy: 1950s–1990s. Int J Radiat Oncol Biol Phys (2007) 69:1484–95. doi: 10.1016/j.ijrobp.2007.05.034 18035211

[54] Bartlett FR Yarnold JR Donovan EM Evans PM Locke I Kirby AM. Multileaf Collimation Cardiac Shielding in Breast Radiotherapy: Cardiac Doses Are Reduced, But at What Cost? Clin Oncol (R Coll Radiol) (2013) 25:690–6. doi: 10.1016/j.clon.2013.09.002 24083961

[55] Raghunathan D Khilji MI Hassan SA Yusuf SW. Radiation-Induced Cardiovascular Disease. Curr Atheroscler Rep (2017) 19:22. doi: 10.1007/s11883-017-0658-x 28315200

[56] Mast ME van Kempen-Harteveld L Heijenbrok MW Kalidien Y Rozema H Jansen WP. Left-Sided Breast Cancer Radiotherapy With and Without Breath-Hold: Does IMRT Reduce the Cardiac Dose Even Further? Radiother Oncol (2013) 108:248–53. doi: 10.1016/j.radonc.2013.07.017 24044804

[57] Osman SO Hol S Poortmans PM Essers M. Volumetric Modulated Arc Therapy and Breath-Hold in Image-Guided Locoregional Left-Sided Breast Irradiation. Radiother Oncol (2014) 112:17–22. doi: 10.1016/j.radonc.2014.04.004 24825176

[58] Nissen HD Appelt AL. Improved Heart, Lung and Target Dose With Deep Inspiration Breath Hold in a Large Clinical Series of Breast Cancer Patients. Radiother Oncol (2013) 106:28–32. doi: 10.1016/j.radonc.2012.10.016 23199652

[59] Lo YC Yasuda G Fitzgerald TJ Urie MM. Intensity Modulation for Breast Treatment Using Static Multi-Leaf Collimators. Int J Radiat Oncol Biol Phys (2000) 46:187–94. doi: 10.1016/s0360-3016(99)00382-x 10656392

[60] Zhu Q Kirova YM Cao L Arsene-Henry A Chen J. Cardiotoxicity Associated With Radiotherapy in Breast Cancer: A Question-Based Review With Current Literatures. Cancer Treat Rev (2018) 68:9–15. doi: 10.1016/j.ctrv.2018.03.008 29777800

[61] Taylor CW Kirby AM. Cardiac Side-Effects From Breast Cancer Radiotherapy. Clin Oncol (R Coll Radiol) (2015) 27(11):621–9. doi: 10.1016/j.clon.2015.06.007 26133462

[62] Cao Y. Off-Tumor Target–Beneficial Site for Antiangiogenic Cancer Therapy? Nat Rev Clin Oncol (2010) 7:604–8. doi: 10.1038/nrclinonc.2010.118 20683436

[63] Witton CJ Reeves JR Going JJ Cooke TG Bartlett JM. Expression of the HER1-4 Family of Receptor Tyrosine Kinases in Breast Cancer. J Pathol (2003) 200:290–7. doi: 10.1002/path.1370 12845624

[64] Dawood S Broglio K Buzdar AU Hortobagyi GN Giordano SH. Prognosis of Women With Metastatic Breast Cancer by HER-2 Status and Trastuzumab Treatment: An Institutional-Based Review. J Clin Oncol (2010) 28:92–8. doi: 10.1200/JCO.2008.19.9844 PMC2799236 19933921

[65] Viani GA Afonso SL Stefano EJ De Fendi LI Soares FV. Adjuvant Trastuzumab in the Treatment of Her-2-Positive Early Breast Cancer: A Meta-Analysis of Published Randomized Trials. BMC Cancer (2007) 7:153. doi: 10.1186/1471-2407-7-153 17686164 PMC1959236

[66] Chin-Yee NJ Yan AT Kumachev A Ko D Earle C Tomlinson G. Association of Hospital and Physician Case Volumes With Cardiac Monitoring and Cardiotoxicity During Adjuvant Trastuzumab Treatment for Breast Cancer: A Retrospective Cohort Study. CMAJ Open (2016) 4:e66–72. doi: 10.9778/cmajo.20150033 PMC4866921 27280116

[67] Arnould L Gelly M Penault-Llorca F Benoit L Bonnetain F Migeon C. Trastuzumab-Based Treatment of HER-2-Positive Breast Cancer: An Antibody-Dependent Cellular Cytotoxicity Mechanism? Br J Cancer (2006) 94:259–67. doi: 10.1038/sj.bjc.6602930 PMC2361112 16404427

[68] Junttila TT Akita RW Parsons K Fields C Lewis Phillips GD Friedman LS. Ligand-Independent HER-2/HER3/PI3K Complex Is Disrupted by Trastuzumab and Is Effectively Inhibited by the PI3K Inhibitor GDC-0941. Cancer Cell (2009) 15:429–40. doi: 10.1016/j.ccr.2009.03.020 19411071

[69] Le XF Claret FX Lammayot A Tian L Deshpande D LaPushin R. The Role of Cyclin-Dependent Kinase Inhibitor p27Kip1 in Anti-HER-2 Antibody-Induced G1 Cell Cycle Arrest and Tumor Growth Inhibition. J Biol Chem (2003) 278:23441–50. doi: 10.1074/jbc.M300848200 12700233

[70] Seidman A Hudis C Pierri MK Shak S Paton V Ashby M. Cardiac Dysfunction in the Trastuzumab Clinical Trials Experience. J Clin Oncol (2002) 20:1215–21. doi: 10.1200/JCO.2002.20.5.1215 11870163

[71] Onitilo AA Engel JM Stankowski RV. Cardiovascular Toxicity Associated With Adjuvant Trastuzumab Therapy: Prevalence, Patient Characteristics, and Risk Factors. Ther Adv Drug Saf (2014) 5:154–66. doi: 10.1177/2042098614529603 PMC4110857 25083270

[72] Wu YT Xu Z Zhang K Wu JS Li X Arshad B. Efficacy and Cardiac Safety of the Concurrent Use of Trastuzumab and Anthracycline-Based Neoadjuvant Chemotherapy for HER-2-Positive Breast Cancer: A Systematic Review and Meta-Analysis. Ther Clin Risk Manag (2018) 14:1789–97. doi: 10.2147/TCRM.S176214 PMC6165855 30310287

[73] Tocchetti CG Ragone G Coppola C Rea D Piscopo G Scala S. Detection, Monitoring, and Management of Trastuzumab-Induced Left Ventricular Dysfunction: An Actual Challenge. Eur J Heart Fail (2012) 14:130–7. doi: 10.1093/eurjhf/hfr165 22219501

[74] Ewer MS Vooletich MT Durand JB Woods ML Davis JR Valero V. Reversibility of Trastuzumab-Related Cardiotoxicity: New Insights Based on Clinical Course and Response to Medical Treatment. J Clin Oncol (2005) 23:7820–6. doi: 10.1200/JCO.2005.13.300 16258084

[75] Şendur MA Aksoy S Yorgun H Ozdemir N Yilmaz FM Yazıcı O. Comparison of the Long Term Cardiac Effects Associated With 9 and 52 Weeks of Trastuzumab in HER-2-Positive Early Breast Cancer. Curr Med Res Opin (2015) 31:547–56. doi: 10.1185/03007995.2015.1005834 25586297

[76] Hussain Y Drill E Dang CT Liu JE Steingart RM Yu AF. Cardiac Outcomes of Trastuzumab Therapy in Patients With HER-2-Positive Breast Cancer and Reduced Left Ventricular Ejection Fraction. Breast Cancer Res Treat (2019) 175:239–46. doi: 10.1007/s10549-019-05139-6 PMC6494676 30721443

[77] Bowles EJ Wellman R Feigelson HS Onitilo AA Freedman AN Delate T. Risk of Heart Failure in Breast Cancer Patients After Anthracycline and Trastuzumab Treatment: A Retrospective Cohort Study. J Natl Cancer Inst (2012) 104:1293–305. doi: 10.1093/jnci/djs317 PMC3433392 22949432

[78] Yu AF Mukku RB Verma S Liu JE Oeffinger KC Steingart RM. Cardiac Safety of non-Anthracycline Trastuzumab-Based Therapy for HER-2-Positive Breast Cancer. Breast Cancer Res Treat (2017) 166:241–7. doi: 10.1007/s10549-017-4362-x PMC5647229 28710537

[79] Lynce F Swain SM. Pertuzumab for the Treatment of Breast Cancer. Cancer Invest (2014) 32:430–8. doi: 10.3109/07357907.2014.922570 24921704

[80] Agus DB Akita RW Fox WD Lewis GD Higgins B Pisacane PI. Targeting Ligand-Activated ErbB2 Signaling Inhibits Breast and Prostate Tumor Growth. Cancer Cell (2002) 2:127–37. doi: 10.1016/s1535-6108(02)00097-1 12204533

[81] Baselga J Cortés J Kim SB Im SA Hegg R Im YH. Pertuzumab Plus Trastuzumab Plus Docetaxel for Metastatic Breast Cancer. N Engl J Med (2012) 366:109–19. doi: 10.1056/NEJMoa1113216 PMC5705202 22149875

[82] Lenihan D Suter T Brammer M Neate C Ross G Baselga J. Pooled Analysis of Cardiac Safety in Patients With Cancer Treated With Pertuzumab. Ann Oncol (2012) 23:791–800. doi: 10.1093/annonc/mdr294 21665955 PMC3331733

[83] Swain SM Ewer MS Cortés J Amadori D Miles D Knott A. Cardiac Tolerability of Pertuzumab Plus Trastuzumab Plus Docetaxel in Patients With HER-2-Positive Metastatic Breast Cancer in CLEOPATRA: A Randomized, Double-Blind, Placebo-Controlled Phase III Study. Oncologist (2013) 18:257–64. doi: 10.1634/theoncologist.2012-0448 PMC3607520 23475636

[84] Schneeweiss A Chia S Hickish T Harvey V Eniu A Hegg R. Pertuzumab Plus Trastuzumab in Combination With Standard Neoadjuvant Anthracycline-Containing and Anthracycline-Free Chemotherapy Regimens in Patients With HER-2-Positive Early Breast Cancer: A Randomized Phase II Cardiac Safety Study (TRYPHAENA). Ann Oncol (2013) 24:2278–84. doi: 10.1093/annonc/mdt182 23704196

[85] Burris HA 3rd. Dual Kinase Inhibition in the Treatment of Breast Cancer: Initial Experience With the EGFR/ErbB-2 Inhibitor Lapatinib. Oncologist (2004) 9 Suppl 3:10–5. doi: 10.1634/theoncologist.9-suppl_3-10 15163842

[86] Perez EA Koehler M Byrne J Preston AJ Rappold E Ewer MS. Cardiac Safety of Lapatinib: Pooled Analysis of 3689 Patients Enrolled in Clinical Trials. Mayo Clin Proc (2008) 83:679–86. doi: 10.4065/83.6.679 18533085

[87] Morris PG Iyengar NM Patil S Chen C Abbruzzi A Lehman R. Long-Term Cardiac Safety and Outcomes of Dose-Dense Doxorubicin and Cyclophosphamide Followed by Paclitaxel and Trastuzumab With and Without Lapatinib in Patients With Early Breast Cancer. Cancer (2013) 119:3943–51. doi: 10.1002/cncr.28284 24037735

[88] Eiger D Pondé NF Agbor-Tarh D Moreno-Aspitia A Piccart M Hilbers FS. Long-Term Cardiac Outcomes of Patients With HER-2-Positive Breast Cancer Treated in the Adjuvant Lapatinib and/or Trastuzumab Treatment Optimization Trial. Br J Cancer (2020) 122:1453–60. doi: 10.1038/s41416-020-0786-x PMC7217956 32203207

[89] Dogan E Yorgun H Petekkaya I Ozer N Altundag K Ozisik Y. Evaluation of Cardiac Safety of Lapatinib Therapy for ErbB2-Positive Metastatic Breast Cancer: A Single Center Experience. Med Oncol (2012) 29:3232–9. doi: 10.1007/s12032-012-0253-5 22729366

[90] Hasinoff BB Patel D Wu X. The Dual-Targeted HER1/HER-2 Tyrosine Kinase Inhibitor Lapatinib Strongly Potentiates the Cardiac Myocyte-Damaging Effects of Doxorubicin. Cardiovasc Toxicol (2013) 13:33–47. doi: 10.1007/s12012-012-9183-x 22948710

[91] Hug B Abbas R Leister C Burns J Sonnichsen D. A Single-Dose, Crossover, Placebo- and Moxifloxacin-Controlled Study to Assess the Effects of Neratinib (HKI-272) on Cardiac Repolarization in Healthy Adult Subjects. Clin Cancer Res (2010) 16:4016–23. doi: 10.1158/1078-0432.CCR-10-0280 20647478

[92] Cadeddu C Piras A Dessì M Madeddu C Mantovani G Scartozzi M. Timing of the Negative Effects of Trastuzumab on Cardiac Mechanics After Anthracycline Chemotherapy. Int J Cardiovasc Imaging (2017) 33:197–207. doi: 10.1007/s10554-016-0987-9 27696298

[93] Suter TM Cook-Bruns N Barton C. Cardiotoxicity Associated With Trastuzumab (Herceptin) Therapy in the Treatment of Metastatic Breast Cancer. Breast (2004) 13:173–83. doi: 10.1016/j.breast.2003.09.002 15177418

[94] Huang C Zhang X Ramil JM Rikka S Kim L Lee Y. Juvenile Exposure to Anthracyclines Impairs Cardiac Progenitor Cell Function and Vascularization Resulting in Greater Susceptibility to Stress-Induced Myocardial Injury in Adult Mice. Circulation (2010) 121:675–83. doi: 10.1161/CIRCULATIONAHA.109.902221 PMC2834271 20100968

[95] Molinaro M Ameri P Marone G Petretta M Abete P Di Lisa F. Recent Advances on Pathophysiology, Diagnostic and Therapeutic Insights in Cardiac Dysfunction Induced by Antineoplastic Drugs. BioMed Res Int (2015) 2015:138148. doi: 10.1155/2015/138148 26583088 PMC4637019

[96] Odiete O Hill MF Sawyer DB. Neuregulin in Cardiovascular Development and Disease. Circ Res (2012) 111:1376–85. doi: 10.1161/CIRCRESAHA.112.267286 PMC3752394 23104879

[97] Riccio G Esposito G Leoncini E Contu R Condorelli G Chiariello M. Cardiotoxic Effects, or Lack Thereof, of Anti-ErbB2 Immunoagents. FASEB J (2009) 23:3171–8. doi: 10.1096/fj.09-131383 19417081

[98] Milano G Raucci A Scopece A Daniele R Guerrini U Sironi L. Doxorubicin and Trastuzumab Regimen Induces Biventricular Failure in Mice. J Am Soc Echocardiogr (2014) 27:568–79. doi: 10.1016/j.echo.2014.01.014 24534652

[99] De Keulenaer GW Doggen K Lemmens K. The Vulnerability of the Heart as a Pluricellular Paracrine Organ: Lessons From Unexpected Triggers of Heart Failure in Targeted ErbB2 Anticancer Therapy. Circ Res (2010) 106:35–46. doi: 10.1161/CIRCRESAHA.109.205906 20056944

[100] Gradishar WJ Anderson BO Abraham J Aft R Agnese D Allison KH. Breast Cancer, Version 3.2020, NCCN Clinical Practice Guidelines in Oncology. J Natl Compr Canc Netw (2020) 18:452–78. doi: 10.6004/jnccn.2020.0016 32259783

[101] Murphy CG Dickler MN. The Role of CDK4/6 Inhibition in Breast Cancer. Oncologist (2015) 20:483–90. doi: 10.1634/theoncologist.2014-0443 PMC4425391 25876993

[102] Kim ES Scott LJ. Palbociclib: A Review in HR-Positive, HER-2-Negative, Advanced or Metastatic Breast Cancer. Target Oncol (2017) 12:373–83. doi: 10.1007/s11523-017-0492-7 28488183

[103] Santoni M Occhipinti G Romagnoli E Miccini F Scoccia L Giulietti M. Different Cardiotoxicity of Palbociclib and Ribociclib in Breast Cancer: Gene Expression and Pharmacological Data Analyses, Biological Basis, and Therapeutic Implications. BioDrugs (2019) 33:613–20. doi: 10.1007/s40259-019-00382-1 31529317

[104] Wang Z Li J Wang Y Liu Q. Palbociclib Improves Cardiac Dysfunction in Diabetic Cardiomyopathy by Regulating Rb Phosphorylation. Am J Transl Res (2019) 11:3481–9. PMC6614619 31312360

[105] Caulfield SE Davis CC Byers KF. Olaparib: A Novel Therapy for Metastatic Breast Cancer in Patients With a BRCA1/2 Mutation. J Adv Pract Oncol (2019) 10:167–74. doi: 10.6004/jadpro.2019.10.2.6 PMC6750920 31538027

[106] Godet I Gilkes DM. BRCA1 and BRCA2 Mutations and Treatment Strategies for Breast Cancer. Integr Cancer Sci Ther (2017) 4:1–7. doi: 10.15761/ICST.1000228 PMC5505673 28706734

[107] Griguolo G Dieci MV Guarneri V Conte P. Olaparib for the Treatment of Breast Cancer. Expert Rev Anticancer Ther (2018) 18:519–30. doi: 10.1080/14737140.2018.1458613 29582690

[108] Helen S Ruth P Karen S Sally G Wendy B Marc-Antoine F. Olaparib Does Not Cause Clinically Relevant QT/QTc Interval Prolongation in Patients With Advanced Solid Tumours: Results From Two Phase I Studies. Cancer Chemother Pharmacol (2016) 78:775–84. doi: 10.1007/s00280-016-3124-5 27553432

[109] Korkmaz-Icöz S Szczesny B Marcatti M Li S Ruppert M Lasitschka F. Olaparib Protects Cardiomyocytes Against Oxidative Stress and Improves Graft Contractility During the Early Phase After Heart Transplantation in Rats. Br J Pharmacol (2018) 175:246–61. doi: 10.1111/bph.13983 PMC5758401 28806493

[110] Wander SA Hennessy BT Slingerland JM. Next-Generation mTOR Inhibitors in Clinical Oncology: How Pathway Complexity Informs Therapeutic Strategy. J Clin Invest (2011) 121:1231–41. doi: 10.1172/JCI44145 PMC3069769 21490404

[111] Lebwohl D Thomas G Lane HA O’Reilly T Escudier B Yao JC. Research and Innovation in the Development of Everolimus for Oncology. Expert Opin Drug Discov (2011) 6:323–38. doi: 10.1517/17460441.2011.558079 22647206

[112] Rugo HS Pritchard KI Gnant M Noguchi S Piccart M Hortobagyi G. Incidence and Time Course of Everolimus-Related Adverse Events in Postmenopausal Women With Hormone Receptor-Positive Advanced Breast Cancer: Insights From BOLERO-2. Ann Oncol (2014) 25:808–15. doi: 10.1093/annonc/mdu009 PMC3969554 24615500

[113] Lim YP Lin CL Lin YN Ma WC Hung DZ Kao CH. Tamoxifen Treatment and the Reduced Risk of Hyperlipidemia in Asian Patients With Breast Cancer: A Population-Based Cohort Study. Clin Breast Cancer (2015) 15:294–300. doi: 10.1016/j.clbc.2015.03.005 25922283

[114] Victor GV. The NSABP Study of Tamoxifen and Raloxifene (STAR) Trial. Expert Rev Anticancer Ther (2009) 9:51–60. doi: 10.1586/14737140.9.1.51 19105706 PMC2785111

[115] Nordenskjöld B Rosell J Rutqvist LE Malmström PO Bergh J Bengtsson NO. Coronary Heart Disease Mortality After 5 Years of Adjuvant Tamoxifen Therapy: Results From a Randomized Trial. J Natl Cancer Inst (2005) 97:1609–10. doi: 10.1093/jnci/dji342 16264181

[116] Henderson VW Lobo RA. Hormone Therapy and the Risk of Stroke: Perspectives 10 Years After the Women’s Health Initiative Trials. Climacteric (2012) 15:229–34. doi: 10.3109/13697137.2012.656254 PMC3675220 22612608

[117] Reis SE Costantino JP Wickerham DL Tan-Chiu E Wang J Kavanah M. Cardiovascular Effects of Tamoxifen in Women With and Without Heart Disease: Breast Cancer Prevention Trial. J Natl Cancer Inst (2001) 93:16–21. doi: 10.1093/jnci/93.1.16 11136837

[118] Vogel CL Johnston MA Capers C Braccia D. Toremifene for Breast Cancer: A Review of 20 Years of Data. Clin Breast Cancer (2014) 14:1–9. doi: 10.1016/j.clbc.2013.10.014 24439786

[119] Deeks ED. Fulvestrant: A Review in Advanced Breast Cancer Not Previously Treated With Endocrine Therapy. Drugs (2018) 78:131–7. doi: 10.1007/s40265-017-0855-5 29270913

[120] Di Leo A Johnston S Lee KS Ciruelos E Lønning PE Janni W. Buparlisib Plus Fulvestrant in Postmenopausal Women With Hormone-Receptor-Positive, HER-2-Negative, Advanced Breast Cancer Progressing on or After mTOR Inhibition (BELLE-3): A Randomised, Double-Blind, Placebo-Controlled, Phase 3 Trial. Lancet Oncol (2018) 19:87–100. doi: 10.1016/S1470-2045(17)30688-5 29223745

[121] Robertson JF. Fulvestrant (Faslodex) – How to Make a Good Drug Better. Oncologist (2007) 12:774–84. doi: 10.1634/theoncologist.12-7-774 17673609

[122] Rocca A Maltoni R Bravaccini S Donati C Andreis D. Clinical Utility of Fulvestrant in the Treatment of Breast Cancer: A Report on the Emerging Clinical Evidence. Cancer Manag Res (2018) 10:3083–99. doi: 10.2147/CMAR.S137772 PMC6124791 30214302

[123] Breast International Group (BIG) 1-98 Collaborative Group Thürlimann B Keshaviah A Coates AS Mouridsen H Mauriac L. A Comparison of Letrozole and Tamoxifen in Postmenopausal Women With Early Breast Cancer. N Engl J Med (2005) 353:2747–57. doi: 10.1056/NEJMoa052258 16382061

[124] Coates AS Keshaviah A Thürlimann B Mouridsen H Mauriac L Forbes JF. Five Years of Letrozole Compared With Tamoxifen as Initial Adjuvant Therapy for Postmenopausal Women With Endocrine-Responsive Early Breast Cancer: Update of Study BIG 1-98. J Clin Oncol (2007) 25:486–92. doi: 10.1200/JCO.2006.08.8617 17200148

[125] Amir E Seruga B Niraula S Carlsson L Ocaña A. Toxicity of Adjuvant Endocrine Therapy in Postmenopausal Breast Cancer Patients: A Systematic Review and Meta-Analysis. J Natl Cancer Inst (2011) 103:1299–309. doi: 10.1093/jnci/djr242 21743022

[126] Kemp A Preen DB Saunders C Boyle F Bulsara M Holman CD. Women Commencing Anastrozole, Letrozole or Tamoxifen for Early Breast Cancer: The Impact of Comorbidity and Demographics on Initial Choice. PloS One (2014) 9:e84835. doi: 10.1371/journal.pone.0084835 24392158 PMC3879327

[127] Shekarforoush S Koohpeyma F Safari F. Alteration at Transcriptional Level of Cardiac Renin-Angiotensin System by Letrozole Treatment. Acta Cardiol (2019) 74:109–13. doi: 10.1080/00015385.2018.1472840 29909753

[128] Kaufman B Mackey JR Clemens MR Bapsy PP Vaid A Wardley A. Trastuzumab Plus Anastrozole Versus Anastrozole Alone for the Treatment of Postmenopausal Women With Human Epidermal Growth Factor Receptor 2-Positive, Hormone Receptor-Positive Metastatic Breast Cancer: Results From the Randomized Phase III TAnDEM Study. J Clin Oncol (2009) 27:5529–37. doi: 10.1200/JCO.2008.20.6847 19786670

[129] Cuzick J Sestak I Forbes JF Dowsett M Cawthorn S Mansel RE. Use of Anastrozole for Breast Cancer Prevention (IBIS-II): Long-Term Results of a Randomised Controlled Trial. Lancet (2020) 395:117–22. doi: 10.1016/S0140-6736(19)32955-1 PMC6961114 31839281

[130] Armenian SH Lacchetti C Barac A Carver J Constine LS Denduluri N. Prevention and Monitoring of Cardiac Dysfunction in Survivors of Adult Cancers: American Society of Clinical Oncology Clinical Practice Guideline. J Clin Oncol (2017) 35:893–911. doi: 10.1200/JCO.2016.70.5400 27918725

[131] Zamorano JL Lancellotti P Rodriguez Munoz D Aboyans V Asteggiano R Galderisi M. ESC Scientific Document Group 2016 ESC Position Paper on Cancer Treatments and Cardiovascular Toxicity Developed Under the Auspices of the ESC Committee for Practice Guidelines: The Task Force for Cancer Treatments and Cardiovascular Toxicity of the European Society of Cardiology (ESC). Eur Heart J (2016) 37:2768–801. doi: 10.1093/eurheartj/ehw211 27567406

